# Beyond Motor Noise: Considering Other Causes of Impaired Reinforcement Learning in Cerebellar Patients

**DOI:** 10.1523/ENEURO.0458-18.2019

**Published:** 2019-02-22

**Authors:** Pierre Vassiliadis, Gerard Derosiere, Julie Duque

**Affiliations:** Institute of Neuroscience, Université Catholique de Louvain, Brussels, Belgium 1200

**Keywords:** basal ganglia, cerebellum, motor learning, motor noise, reinforcement learning

## Significance Statement

Motor and reinforcement learning have been classically linked to functionally independent brain networks centered on the cerebellum and the basal ganglia, respectively. In a recent study published in *eNeuro*, Therrien et al. (2018) showed that increasing motor noise in healthy subjects disrupts reinforcement learning. However, this impairment remained well below that detected in cerebellar patients even when motor noise in healthy subjects was adjusted to match that observed in the patients. This suggests that impaired reinforcement learning following cerebellar damage cannot be solely accounted for by altered motor noise in these patients. Based on recent anatomic and functional evidence, we argue that the cerebellum may directly contribute to reinforcement learning, consistent with its tight connections with the basal ganglia.

## 

The ability to adapt to changes occurring in the environment is a fundamental feature of human behavior, which relies on both sensory and reward feedbacks. On the one hand, the role of sensory feedback has been largely considered by studying how motor commands adapt to visual perturbations (e.g., a visuomotor rotation), a process called error-based learning (Shadmehr et al., 2010; Wolpert et al., 2011; Kim et al., 2018; Roemmich and Bastian, 2018). This type of motor learning involves the computation of sensory prediction errors (SPEs), namely, the difference between predicted and actual sensory outcome (Tseng et al., 2007; Schlerf and Ivry, 2012; Shadmehr, 2017, 2018). On the other hand, the role of reward feedback has been mostly investigated in tasks that require learning what action to select or not, by updating reward predictions based on previous experience, a process named reinforcement learning (Lee et al., 2012; Derosiere et al., 2017a,b; Gershman and Daw, 2017; O’Doherty et al., 2017). A central aspect here is the computation of reward prediction errors (RPEs), namely, the difference between predicted and actual rewards (Schultz, 2015).

For a long time, motor learning and reinforcement learning have been studied apart and have been linked to functionally independent brain networks (Doya, 2000), mostly centered on either the cerebellum (Tseng et al., 2007; Schlerf and Ivry, 2012; Taylor and Ivry, 2014; Herzfeld et al., 2018) or on dopaminergic-basal ganglia circuits (Lee et al., 2012; O’Doherty et al., 2017), respectively. However, this view has changed in the past few years, with recent works indicating that rewards can strongly impact motor learning (Abe et al., 2011; Izawa and Shadmehr, 2011; Dayan et al., 2014; Galea et al., 2015; Nikooyan et al., 2015; Quattrocchi et al., 2017; Song and Smiley-Oyen, 2017). Hence, efforts are now made to understand how motor and reinforcement learning may interact at the neural level (Wilkinson et al., 2015; Mawase et al., 2017; Uehara et al., 2017). Consistently, Therrien et al. (2016) recently reported data pointing toward an implication of the cerebellum in reinforcement-based motor learning. As such, cerebellar patients exhibited a reduced ability to learn from reinforcement in a visuomotor adaptation task, compared to healthy subjects (Therrien et al., 2016). However, because the patients also exhibited increased motor noise (i.e., defined as an uncontrollable source of motor variability), this deficit could have occurred indirectly, due to an impaired ability to precisely relate an action to the received reward. The work reported by the same authors in *eNeuro* aimed at tackling this issue by increasing motor noise artificially in healthy individuals (Therrien et al., 2018).

Therrien et al. (2018) used the same visuomotor adaptation task as in their previous 2016 paper. Specifically, the healthy subjects were required to make reaching movements toward a visual target with no visual information on the position of their hand and received binary reward feedback. To get a reward, subjects had to learn to alter their reach angle to counteract a visuomotor rotation between the location of the visual target and their hand position: the reward feedback was based on this modified reach angle. Critically, the authors added motor noise by deviating the subjects’ reach in a way that was proportional to baseline motor variability. The experimental design involved both a low and a high noise condition, with the latter set to approximate the level of motor noise observed in cerebellar patients (Therrien et al., 2016). Hence, such approach allowed comparing reinforcement learning abilities between patients and healthy controls with comparable motor noise. The authors report an impaired reinforcement learning in healthy individuals in the high-noise compared to the control condition (i.e., when no noise was added). Yet, one critical result is that this impairment remained well below that observed in cerebellar patients. This finding indicates that motor noise does not entirely account for the reinforcement learning deficits observed following cerebellar damage.

A main line of argumentation in the paper focuses on the reduced proprioceptive acuity of cerebellar patients. As such, even with added motor noise, healthy subjects can still relate the rewards they receive to their reach angle, based on proprioception, while this ability is known to be altered in cerebellar patients (Miall and King, 2008; Bhanpuri et al., 2013; Weeks et al., 2017a,b). Hence, the reduction in proprioceptive precision might have indirectly altered reinforcement learning in the patients. Note though that the clinical tests run by Therrien et al. (2016) failed to reveal any reduction in proprioceptive precision in the patients. Hence, even if a discrete reduction in proprioceptive precision could have gone unnoticed based on clinical tests (Rinderknecht et al., 2018), we would like to propose that alterations in proprioceptive precision may not completely explain reinforcement learning deficits observed in the patients. Rather, cerebellar damage may directly alter reinforcement learning, as already suggested by others (Swain et al., 2011; McDougle et al., 2016; Miall and Galea, 2016).

Our viewpoint is supported by recent anatomic studies showing bidirectional connections between the cerebellum and dopaminergic-basal ganglia routes (Bostan et al., 2010; Chen et al., 2014; Bostan and Strick, 2018). Specifically, the dentate nucleus of the cerebellum sends disynaptic projections to the striatum (Hoshi et al., 2005), and to midbrain dopaminergic structures (Watabe-Uchida et al., 2012). Conversely, the cerebellar cortex receives disynaptic projections from the subthalamic nucleus (Bostan et al., 2010). Functionally, recent works in rodents provide evidence that reward expectation modulates the firing rate of cerebellar cells (Ohmae and Medina, 2015; Wagner et al., 2017; Heffley et al., 2018). In the same vein, neuroimaging studies in humans have reported activity related to RPEs in the cerebellum (O’Doherty, 2004; Ramnani et al., 2004; Seymour et al., 2004; Tanaka et al., 2004; Tobler et al., 2006; Garrison et al., 2013), suggesting that this structure is functionally involved in processing reward feedback. These works are in agreement with the result of a previous study showing that cerebellar patients exhibit altered reinforcement learning in a decision-making task requiring very simple movements (Thoma et al., 2008). In line with these considerations, structural and functional alterations of the cerebellum were found in individuals suffering from an addiction, such as alcohol or cocaine dependence, a condition characterized by abnormal reward processing (Moulton et al., 2014; Miquel et al., 2016; Moreno-Rius and Miquel, 2017). Furthermore, an important feature of reinforcement-based compared to error-based adaptation is that the former increases trial-to-trial movement variability, reflecting an exploration process of the environment (Izawa and Shadmehr, 2011; Taylor and Ivry, 2014; Dhawale and Smith, 2017). Following this idea, modeling work in the present study showed that healthy subjects increased motor exploration following unrewarded compared to rewarded trials. This effect was absent in the patients reflecting an inability to modulate behavior optimally according to reward feedback. In this view, a recent study showed that poor performance in a visuomotor adaptation task in cerebellar patients is not only due to impaired error-based learning but also to a difficulty in using feedback information to develop and maintain an explicit aiming strategy (Butcher et al., 2017). Hence, it seems that cerebellar dysfunction could have impaired the ability to learn both from error and reward feedbacks.

Nevertheless, an important point that needs to be raised here is the age difference between the healthy subjects tested in the commented paper (Therrien et al., 2018) and the cerebellar patients to which they are compared but that were originally tested in Therrien et al. (2016). As such, the healthy subjects (25.0 ± 4.8 years old) were much younger than the patients (61.5 ± 10.0 years old), and in fact, when the groups were matched for age in Therrien et al. (2016), the healthy (older) controls also exhibited impaired motor exploration, to a comparable extent as the patients. This suggests that aging could also have contributed to the reduced reinforcement learning abilities of the patients (Chowdhury et al., 2013). Further studies are therefore required to examine the respective contribution of aging and cerebellar dysfunction to reinforcement learning.

In conclusion, the work by Therrien and colleagues provides new insights into the influence of motor noise on reinforcement learning in healthy subjects and in patients suffering from cerebellar impairment. Moreover, the data are also consistent with the view that the cerebellum may be directly involved in reinforcement learning and more precisely in reinforcement-based motor learning. Future studies could directly test this hypothesis by relating the reinforcement learning impairment of patients to their score at the International Cooperative Ataxia Rating Scale, reflecting the severity of the cerebellar impairment. This line of research opens very interesting perspectives to design innovative multi-approach neurorehabilitation strategies.

## Synthesis

Reviewing Editor: Christian Hansel, University of Chicago Decisions are customarily a result of the Reviewing Editor and the peer reviewers coming together and discussing their recommendations until a consensus is reached. When revisions are invited, a fact-based synthesis statement explaining their decision and outlining what is needed to prepare a revision will be listed below. The following reviewer(s) agreed to reveal their identity: NONE.

In the current manuscript commenting the paper by Therrien et al., on eNeuro (2018 Aug 13; 5(3)) the authors argue that the cerebellum may directly contribute to reinforcement learning. This is an interesting comment that adds value to the paper by Therrien et al. and connects it to anatomical studies showing connections of the cerebellum with areas relevant for reinforcement learning, and functional data on cerebellar firing rate modulation in response to reward expectation.

In this Commentary article on the recently accepted manuscript by Therrien et al., on eNeuro (2018 Aug 13; 5(3)) the authors argue that the cerebellum may directly contribute to reinforcement learning. The current and previous work by Therrien et al. showed that cerebellar patients exhibit a reduced reinforcement learning in a visuomotor adaptation task in association with increased motor noise but, artificially increased motor noise at high-levels in healthy subjects (similar to the level observed in cerebellar patients), caused an impairment in reinforcement learning that remained well below that observed in cerebellar patients. The authors of the manuscript, therefore, argue that motor noise does not entirely account for the deficits in reinforcement learning observed in cerebellar patients suggesting (as already suggested by others) that cerebellar damage may directly alter reinforcement learning. This is supported by previous anatomical studies showing connections of the cerebellum with areas relevant for reinforcement learning, and functional data on cerebellar firing rate modulation in response to reward expectation.   The commentary is well-written and sound in its argumentation. No major revisions are suggested.  It might be relevant to briefly comment on the possible implications for addiction or on possible studies relating alterations in addiction behavior and cerebellar function.   Some additional critical notes on possible weaknesses on the commented paper by Therrien et al. may also be beneficial. For instance, the authors might consider if it is relevant to mention that groups of healthy subjects were apparently not aged-matched with cerebellar patients or that inter-subject variability of ataxia ICARS scores was not considered as a possible factor to be related to the different levels of impairment in reinforcement learning.
